# Sudden Infant Death Syndrome, Pulmonary Edema, and Sodium Toxicity: A Grounded Theory

**DOI:** 10.3390/diseases10030059

**Published:** 2022-08-30

**Authors:** Ronald B. Brown

**Affiliations:** School of Public Health Sciences, University of Waterloo, Waterloo, ON N2L 3G1, Canada; r26brown@uwaterloo.ca

**Keywords:** sudden infant death syndrome, SIDS, pulmonary edema, hypoxia, sodium chloride, hypervolemia, fever, alveolar epithelium, microvascular endothelium

## Abstract

Sudden Infant Death Syndrome (SIDS) occurs unexpectedly in an otherwise healthy infant with no identifiable cause of death following a thorough investigation. A general hypervolemic state has been identified in SIDS, and fluid in the lungs suggests the involvement of pulmonary edema and hypoxia as the cause of death. The present perspective paper reviews pathophysiological, epidemiological, and dietary evidence in SIDS. A grounded theory is presented that proposes an association of SIDS with sodium toxicity from excessive sodium chloride intake, mediated by noncardiogenic pulmonary edema, hypoxia, and alveolar damage. The peak of SIDS cases occurs in infants 2–4 months of age, who are less efficient in excreting excessive dietary sodium load. Evidence implicating sodium toxicity in SIDS includes increased levels of sodium associated with fever and with inflammatory/immune responses in the lungs. Conditions in near-miss SIDS cases are linked to dysregulated sodium, and increased sodium dietary intake suggests that sodium toxicity from a high-salt diet potentially mediates the association of seasonality and socioeconomic status with SIDS incidence. In addition, exposure to sodium toxicity meets three main criteria of the triple risk model of SIDS. The proposed pathophysiological effects of pulmonary edema related to sodium toxicity in SIDS merit further investigations.

## 1. Introduction

Sudden infant death syndrome (SIDS) is currently defined by the World Health Organization (WHO) as “the abrupt and unexplained death of an apparently healthy infant under one year of age, remaining unexplained after a thorough case investigation, including performance of a complete autopsy, examination of the death scene, and review of the clinical history” [1]. Accidental suffocation/asphyxia and deaths due to uncertain circumstances are listed separately from SIDS, under sudden unexpected infant death (SUID) [2]. This diagnostic shift reassigns asphyxia or undetermined causes to the sleep environment, which “serves to underestimate the actual mortality of what was once considered SIDS” [3].

Autopsy findings of SIDS cases include increased brain weight, possibly caused by cerebral edema “secondary to hypoxia/anoxia or toxic/metabolic factors” [4]. However, further postmortem examinations suggest that environmental toxins, including lead, mercury, and arsenic, are not a cause of SIDS [5]. On the other hand, sodium toxicity, the toxic effect from acute salt poisoning [6] or from prolonged intake of excessive dietary sodium [7], is an unexamined toxic factor potentially involved in SIDS.

Excessive dietary salt intake (sodium chloride) is strongly associated with hypervolemia, fluid overload [8], and blood–brain barrier dysregulation [9]. Of relevance, “abnormalities of regulation of the blood brain barrier with disturbances in water homeostasis” could contribute to increased brain weight in SIDS, although possible causes remain controversial [10]. Other organs with increased weight in SIDS include the thymus, liver, and lungs [4]. “It is clear, however, that in SIDS some of these organs are fluid laden and thus heavy” [11]. Importantly, congestion of organs in SIDS is part of a general hypervolemic state of the total body from excess fluid volume [12].

Excessive fluid in the lungs from noncardiogenic pulmonary edema causes acute hypoxia [13], known as acute-onset hypoxemic respiratory failure (AHRF) [14], which may be a causative factor in SIDS. Furthermore, high-permeability pulmonary edema (HPPE) [15] due to injury of the alveolar capillary basement membrane may be associated with pulmonary intra-alveolar hemorrhage in SIDS [16]. “Intra-alveolar haemorrhage would contribute to lung heaviness” in SIDS, caused by “leakage of fluid into the alveolae and interstitium” [11]. Pulmonary lymphatic stasis associated with pulmonary edema has been observed in SIDS [17], and “lung lymphatics play an important role in the interstitial edema clearance” [18].

## 2. Method

This perspective paper reviews pathophysiological, epidemiological, and dietary evidence with respect to SIDS and synthesizes new insights, proposing an association between SIDS and sodium toxicity from excessive sodium chloride intake mediated by noncardiogenic pulmonary edema, hypoxia, and alveolar damage. A grounded theory method was used to rigorously and objectively search and review relevant concepts from the research literature on SIDS [19]. Keywords searched included “sudden infant death syndrome”, “dietary sodium”, “pulmonary edema”, “hypoxia”, and “alveolar epithelium”. Additional keywords from studies cited in the retrieved literature were searched as well. Using an iterative process of comparative analysis, concepts from the selected research literature were formed into themes, then themes were developed into relationships in order to induce an explanatory theory that proposes a causative link between sodium toxicity and SIDS. The grounded theory in this paper provides novel insights and new directions for further research and hypothesis testing in the etiology of SIDS and its potential relationship with the toxic effects of excessive sodium levels in infants.

## 3. Alveolar Epithelial Permeability

**Figure 1:**
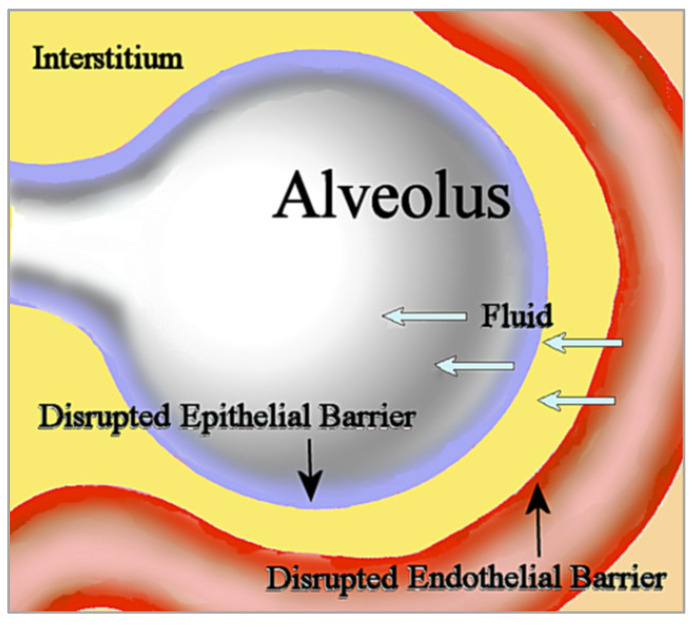
In noncardiogenic pulmonary edema, fluid flows into the interstitium through the disrupted microvascular endothelial barrier, then subsequently flows into the alveolus through the disrupted epithelial barrier.

Noncardiogenic pulmonary edema is unrelated to the increased vascular hydrostatic pressure in cardiogenic pulmonary edema usually seen in heart failure. In noncardiogenic pulmonary edema, disruption of the microvascular endothelial barrier increases permeability to fluid, which then fills the interstitium. Disruption of the alveolar epithelial barrier additionally increases permeability to interstitial fluid that floods the alveolus [20]; see Figure 1. This influx of edematous fluid may contain red blood cells [21].

Importantly, “resolution of alveolar oedema depends on the active removal of salt and water from the distal air spaces of the lung across the distal lung epithelial barrier” [22]. Salt water aspiration is a well-known cause of acute salt water-induced lung injury, and produces severe pulmonary edema, hypoxia, inflammation, apoptosis, and oxidative stress [23,24]. Of relevance, rapid administration of intravenous 0.9% saline (sodium chloride) can cause lung injury and interstitial permeability pulmonary edema in healthy volunteers [25], and acute and chronic salt load from intravenous hypertonic saline infusions in healthy participants in a randomized intervention increased microvascular permeability, with “direct effects on the endothelial surface layer” [26].

High dietary salt impairs endothelial function in rodents and in healthy normotensive humans independent of blood pressure and hypertension, a vascular effect that researchers have attributed to oxidative stress and damage to the endothelial glycocalyx (eGCX) [27]. The eGCX is a coating on the epithelial cell that normally maintains endothelial barrier integrity and regulates cell permeability; “eGCX degradation products act as pathogenic factors capable of inducing endothelial hyperpermeability and microvascular leakage during inflammation” [28]. The combination of intense inflammatory response and leukocyte infiltration within alveoli is associated with injury to the alveolar–epithelial barrier, leading to pulmonary edema in acute lung injury [29]:

“The finding of greater numbers of T lymphocytes, B lymphocytes, and eosinophils in the lungs in SIDS compared to controls suggests that an abnormal or inappropriate inflammatory response had occurred in association with SIDS, an accumulation consistent with expression of the cytokines interleukin 4, interleukin 5, and granulocyte-macrophage colony-stimulating factor which are associated with TH2 helper cell phenotype”

[30]

Of relevance, sodium chloride intake is associated with immune-mediated inflammatory responses that increase T-cell proliferation of pro-inflammatory M1 macrophages while suppressing anti-inflammatory M2 macrophages [31]. Sodium chloride exposure has been found to enhance the production of cytokines, including interleukin 4 and interleukin 5 [32], and high salt levels increase production of granulocyte–macrophage colony-stimulating factor, which is associated with a pathogenic phenotype of Th-17 cells [33]. Furthermore, leukocytosis has been induced in rats injected with saline [34], and earlier research postulated that sodium regulates white blood cell counts [35], inferring a potential link between sodium toxicity and greater numbers of eosinophils and lymphocytes in the lungs in SIDS.

**Figure 2:**
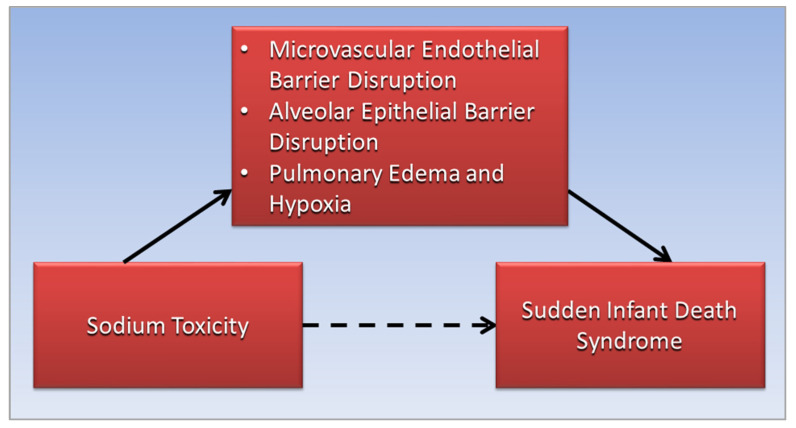
The association of sodium toxicity with sudden infant death syndrome (dotted arrow) is mediated (straight arrows) by disruptions of the microvascular endothelial and alveolar epithelial barriers in the lungs, leading to pulmonary edema and hypoxia (straight arrows).

Figure 2 shows a directed acyclic graph in which the association of sodium toxicity with SIDS is mediated by microvascular endothelial barrier disruption, alveolar epithelial barrier disruption, pulmonary edema, and hypoxia.

## 4. Sodium Channelopathies and SIDS

Impaired contraction of skeletal respiratory muscles, thought to contribute to respiratory failure in SIDS, is caused by variants in the voltage-gated sodium channel NaV1.4, which is encoded by the *SCN4A* gene. A case-control study found that variants of *SCN4A* caused dysfunctional sodium channels in SIDS cases but not in controls [36]. However, the *SCN4A* variants were rare and were found in only 1.4% of SIDS cases, suggesting that impairment of respiratory muscles in SIDS is not the main pathophysiological mechanism involving dysfunctional sodium channels.

Additionally, the NaV1.5 sodium ion channel and subunit variants of the encoding *SCN5A* family of genes are related to “potentially lethal” cardiac arrhythmias in SIDS, but the variants only affect approximately 1% of cases [37]. Taken together, these genetic findings suggest that while sodium toxicity can affect a variety of mechanisms related to dysfunctional sodium channelopathies in SIDS, respiratory failure from pulmonary edema related to sodium toxicity remains under-investigated as a key pathophysiological mechanism in SIDS.

Disturbances in sodium and water homeostasis commonly occur in severely ill neurology patients, and “have a profound effect on the brain” [38]. This effect suggests a potential link between dysfunctional sodium channelopathies in the brain and brainstem alterations thought to affect respiration in SIDS, such as reduced levels of serotonin 5-HT neurotransmitter [39], although more research is needed in this area. Similar to sodium channelopathies in SIDS that affect respiratory muscles and arrhythmias, future studies should investigate whether brainstem alternations in SIDS are a side effect of sodium toxicity rather than the key pathophysiological mechanism leading to respiratory failure.

## 5. Hyperthermia and Fever

Hyperthermia and fever are pathological factors associated with SIDS; the bodies of deceased children with SIDS are often found hot and sweaty, suggesting that environmental conditions that cause hyperthermia, such as overclothing and heavy bedding, may be causative factors in SIDS [40]. However, heat stress is not a direct or significant cause of SIDS [41]. Importantly, whereas hyperthermia related to heat stress is an unregulated rise of core body temperature due to environmental conditions, fever is a regulated increase in internal temperature due to resetting by the hypothalamus, and is often seen in infections [40]. Additionally, “many SIDS infants have a history of viral illness preceding death” [42], and the number of cases of SIDS reported to have had weekly or more frequent “drenching’” sweats was much higher than control cases [43], likely related to fever in respiratory infections [42].

Injection of 0.9% sodium chloride in lab animals acts as a pyrogen that causes “sodium fever” by affecting the hypothalamus [44]. A similar fever has been noted in humans orally administered sodium chloride [45], which in combination with the association of high salt intake and inflammatory-immune responses in infections, supports involvement of sodium toxicity in SIDS. Rather than a direct cause of death from hyperthermia, fever in SIDS may be a side effect of the sodium toxicity that potentially causes pulmonary edema, leading to hypoxia and death. Furthermore, “frothy fluid escaping from the nose and mouth is seen in about half of infants who die from SIDS” [46], which is “suggestive of pulmonary edema” [47]. Of relevance, “fluid and salt restriction” is suggested in order to reduce the risk of pulmonary edema and respiratory distress with impaired respiratory gas exchange in neonates born prematurely [48]. More research is needed to examine sweating and fever in SIDS related to sodium toxicity.

## 6. Intrathoracic Petechial Hemorrhages

Among the distinctive features of SIDS, small blood vessel haemorrhages appear on the surface of the heart, lungs, and thymus, called intrathoracic petechiae; *“*the almost universal finding of intrathoracic petechiae in SIDS stands out as a poorly investigated phenomenon” [49]. Early investigators suggested petechiae in SIDS were caused by respiratory obstruction, although similar petechiae in infants were not found in cases of suffocation or asphyxia [50]. While subpleural and subpericardial hemorrhages are a sign of terminal respiratory obstruction, petechiae in SIDS may be related to other factors. Furthermore, salt induces endothelial cell secretion of the Willebrand factor, which causes hypercoagulability in blood clotting [51], and damage to the endothelial glycocalyx from excessive sodium impairs inhibition of platelet aggregation and adhesion, which “is thought to contribute to thrombosis formation” [52]. These mechanisms imply that intrathoracic petechial hemorrhages in SIDS may be associated with blood clotting in small vessels of the heart, lungs, and thymus from exposure to high salt concentrations. More research is needed in this area.

## 7. Sodium and Near-Miss SIDS

In near-miss SIDS, subsequently categorized as ALTE (apparent life-threatening event) and more recently as BRUE (brief resolved unexplained event) [53], infants rescued from hypoxia where found to have “metabolic acidosis, cardiovascular instability, acute renal failure, ischaemic colitis, or acute neurological dysfunction” [54]. Each of these conditions in near-miss SIDS cases is potentially linked with dysregulated sodium. For example, metabolic acidosis found in rat models of salt-sensitive hypertension was associated with a high-salt diet [55]. Colitis in experiments with mice was exacerbated by a high-salt diet, which was suggested as being caused by inflammatory changes in the gut microbiota [56]. Furthermore, acute kidney injury is exacerbated by excessive salt intake [57], unstable cardiac status is associated with extreme hypernatremia [58], and neurological injury is caused by salt toxicity from rapid ingestion of massive amounts of sodium chloride [59]. These findings provide further support for the involvement of sodium toxicity in SIDS.

## 8. Diet, Sodium, and SIDS

Breastfed infants have lower risk of SIDS compared to non-breastfed infants [60]. Of significance, human breast milk is much lower in sodium compared to cow milk [61], at 15 mg and 43 mg/100 g, respectively [62]. “Infants’ systems cannot handle the high levels of protein, sodium, and potassium of unmodified cow milk”, while infant formula based on modified cow milk “attempts to mimic the nutritional composition of breast milk” [63]. However, according to the World Health Organization, “formula milk marketing, not the product itself, disrupts informed decision-making and undermines breastfeeding and child health” [64]. By comparison, breastfed milk precludes infants’ exposure to sodium salts used in food processing [61,65], which potentially reduces risk of sodium toxicity in breastfed infants compared to formula-fed infants.

As more foods and beverages are gradually introduced into the young child’s diet, intake levels of sodium, calories, and sugar “escalate upward from recommendations in toddlers, becoming even more pronounced among young children” [66]. Note that sodium chloride contains 40% sodium by weight. The World Health Organization recently established new benchmarks to reduce global mean sodium intake by 30%, “with the aim of achieving a target of less than 5 g of salt (i.e., <2 g of sodium) per day by 2025” [67].

Importantly:

“Infants are less efficient than adults at excreting excess sodium, and the sodium intakes of infants should therefore be moderated. By about four months, healthy infants can begin to excrete an excessive sodium load”

[68]

Consequently, increased incidence of SIDS, which peaks between 2–4 months from birth [69], occurs during a developmental period when infants are most vulnerable to harm from excessive sodium intake.

Past feeding studies in the UK found that infants were often introduced to solid foods around three months of age, including “inappropriate use of cows’ milk” and other salty foods, which exceeded daily sodium intake recommendations of 400 mg for infants up to twelve months of age [70]. Earlier research noted that “many parents still make the transition from breast feeding or formula to whole cow’s milk when the infant is less than one year of age” [71]. Research is needed to update statistics on current cow milk consumption in early infancy. Moreover, regarding the COVID-19 pandemic, “health professionals are crucial to successful breastfeeding, but the pandemic could indirectly affect breastfeeding support” [72].

In addition to high sodium levels, cow milk is much higher in phosphorus than breast milk, and excessive dietary phosphate has been linked to degeneration of dopaminergic neurons [73]. Coincidently, a recent study found degeneration of dopaminergic neurons in the majority of 36 SIDS cases, compared to no effect in control infants [74], a finding that is concordant with harm in SIDS from cow milk consumption. Infants’ exposure to excessive minerals in cow milk and other solid foods with potential risk associated with sodium toxicity and SIDS can be reduced by recommendation of exclusive breastfeeding during the first six months after birth, as advised by the American Academy of Pediatrics [75] and the World Health Organization [76].

## 9. Seasonality and Socioeconomic Status in SIDS

Other dietary factors related to SIDS are associated with seasonality and socioeconomic status. Increased seasonal incidence of SIDS is associated with winter, which is concurrent with increased respiratory infections during colder months [77]. However, seasonal SIDS incidence is also concurrent with cold-weather changes in diet. For example, mice increase sodium intake with exposure to cold temperatures [78], and higher estimated salt intake is associated with daytime cold exposure in elderly humans [79]. Studies should investigate infants’ increased sodium intake in winter and potential associations with increased SIDS incidence.

SIDS is more prevalent in populations with lower socioeconomic status (SES), according to an analysis of education, employment, and income factors [80]. Coincidently, dietary sodium intake is higher in children from populations with lower SES as well [81,82], leading to the inference that sodium toxicity is a potential mediating factor that may increase risk of SIDS in populations with lower SES.

## 10. Triple Risk Model and Future Directions

SIDS was first proposed to involve multiple risk factors during the 1970s [83]. The triple risk model, further developed in 1994 by Filiano and Kinney [84], lists three main components of risk in SIDS: a critical developmental period in homeostatic control, exposure to stressors, and underlying susceptibilities. The evidence reviewed in this paper associates sodium toxicity with each of these three main risk factors in the triple risk model of SIDS. The first model component, involving critical development, is consistent with undeveloped kidney function that cannot efficiently regulate high sodium levels in infants before four months of age. The second model component, exposure to stressors, is consistent with pathophysiological stress from sodium toxicity due to exposure to high dietary sodium intake that damages the microvascular endothelial barrier and the alveolar epithelial barrier during pulmonary edema. The third model component, underlying susceptibility to SIDS, is consistent with social determinants that increase an infant’s likelihood of harm due to high sodium intake associated with lower socioeconomic status and heavier salt intake associated with cold seasons. Additional sources of susceptibility to SIDS include genetic determinants, underlying diseases, and conditions that impair sodium regulation or increase toxic effects from high salt intake.

Nutritional epidemiological research is used to study dietary risk factors of diseases within populations [85], and future nutritional epidemiological studies should investigate sodium toxicity from excessive dietary sodium intake in SIDS. Study designs could include randomized controlled clinical trials of a low-sodium dietary intervention to investigate dietary risk reductions in SIDS. Cross-sectional and retrospective case control studies could use food frequency questionnaires to collect dietary information from caregivers and investigate the odds of SIDS associated with high dietary sodium intake. Biomarkers of sodium intake in infants, such as 24-h urinary sodium excretion [86], could contribute valuable data to studying any association between sodium intake and SIDS. Furthermore, SIDS autopsy investigations could report more detailed findings on sodium concentrations in alveoli affected by pulmonary edema.

## 11. Conclusions

The evidence reviewed in this paper supports a novel theory that pulmonary edema is a mediating factor in the association between sodium toxicity and SIDS. Microvascular endothelial barrier permeability and alveolar epithelial barrier permeability from exposure to high levels of sodium in the lungs potentially increases risk of edematous fluid influx, hypoxia, and death. Pulmonary edema and hypervolemia likely account for the heavier weight of lungs and other organs in SIDS cases. Intrathoracic petechial hemorrhages in SIDS may be associated with blood clotting in small vessels of the heart, lungs, and thymus from exposure to high salt concentrations. The peak of SIDS cases occurs in infants 2–4 months of age, who are less efficient in excreting excessive dietary sodium loads. Exclusive breastfeeding for the first six months from birth may protect infants from sodium toxicity associated with SIDS. Other evidence includes increased levels of sodium in fever and inflammatory/immune responses within the lungs. Conditions in near-miss SIDS cases are linked to dysregulated sodium, and increased sodium dietary intake potentially mediates the association of seasonality and socioeconomic status with SIDS incidence. After many decades without a known cause of SIDS, the pathophysiological effects of pulmonary edema and sodium toxicity might provide a missing piece of the puzzle.

## Data Availability

Not applicable.
